# Exploring the potential of *Paris polyphylla* var. *yunnanensis* pollen manipulation in modifying seed dormancy

**DOI:** 10.3389/fpls.2024.1389357

**Published:** 2024-05-22

**Authors:** Meng Wang, Qiuxia Wang, Xiao Wang, Dingkang Wang, Xudong Yin, Yanwen Qiao, Mingkai Ma, Yanli Du, Bin Wang

**Affiliations:** School of Agriculture and Life Sciences, Kunming University, Kunming, Yunnan, China

**Keywords:** *Paris polyphylla* var. *yunnanensis*, preventing anther dehiscence, pattern transmission of expression, dormancy-related genes, anther closure

## Abstract

*Paris polyphylla* var. *yunnanensis*, a well-known Chinese medicinal herb, shows a unique physiological trait characterized by the cyclic opening and closing of its anthers after pollen maturation. The aim of this study was to explore the implications of this phenomenon on breeding. RNA sequencing coupled with methylation sequencing was used to scrutinize and compare gene expression profiles and methylation alterations in pollen and seeds during anther opening and closing, along with cold exposure. Genes enriched within Kyoto Encyclopedia of Genes and Genomes (KEGG) pathways were examined to identify gene clusters susceptible to temperature-related methylation changes in both pollen and seeds. Four pollen treatment models, namely, normal control, “pollen protected from low temperatures,” “pollen from just-opened anther,” and “pollen from close-blocked anther,” were used to produce corresponding seeds via artificial pollination. Subsequently, qRT-PCR was used to validate modifications in the expression patterns of marker genes in pollinated seeds under diverse treatment scenarios. Genes exhibiting significant differences in expression between anthers and normal tissues, along with gene regions linked to methylation variations attributed to low-temperature-treated pollen and seeds, were identified through transcriptomic analysis. Convergence was observed in three signaling pathways: oxidative phosphorylation (ko00190), plant hormone signal transduction (Ko04075), and zeatin biosynthesis (ko00908). Notably, gene clusters prone to temperature-induced methylation changes, such as NADH-ubiquinone oxidoreductase chain 5, plasma membrane ATPase 4, cytochrome c oxidase subunit 2, cis-zeatin O-glucosyltransferase, ABSCISIC ACID-INSENSITIVE 5-like protein 4, and indole-3-acetic acid-amido synthetase (IAAS), were identified. Evaluation using various pollen pollination models revealed altered expression patterns of five dormancy-regulating marker genes: IAAS, sucrose synthase (SUS), gibberellin 2-oxidase (GA2ox), ABA INSENSITIVE 2 (ABI2), and auxin-repressed protein (ARP), in seeds pollinated with pollen from close-blocked anthers, cold-protected pollen, and pollen from freshly opened anthers. The close-blocked anther treatment led to significantly upregulated expression of IAAS, SUS, GA2ox, and ABI2, whereas ARP expression decreased markedly, indicating a propensity toward prolonged seed dormancy. Conversely, in the low-temperature-protected anther model, SUS, ARP, GA2ox, and IAAS exhibited reduced expression levels, whereas the expression of ABI2 was upregulated, overall facilitating seed germination.

## Introduction

*Paris polyphylla* var. *yunnanensis* belongs to the Paris genus of the Trillium family and is widely used as a medicinal herb (Yongbin et al., 2021). This species exhibits low photosynthetic efficiency, and the slow growth of its rhizomes in traditional medicine hinders its rapid propagation (Jiale et al., 2022). The extended growth period from seed planting to harvest, ranging from 6 to 7 years, hampers its artificial propagation, leading to its scarcity as a medicinal resource. Enhancing the efficiency of artificial reproduction in *Paris polyphylla* var. *yunnanensis* remains a critical technological and scientific challenge. The seeds of *Paris polyphylla* var. *yunnanensis* possess physiological characteristics associated with “secondary dormancy” (Yi et al., 2021; Linlin et al., 2022), resulting in low germination rates and reproductive efficiency (Hailan et al., 2018), along with slow resource regeneration (Xiaodong, 2018). Therefore, elucidating the mechanisms and factors that influence seed dormancy is crucial for improving the reproductive efficiency of *Paris polyphylla* var. *yunnanensis*.

Seed dormancy is influenced by the content and proportion of gibberellins (GAs) and abscisic acid (ABA), and the regulation of their synthesis genes is affected by temperature, light, and seed moisture levels during the seed stage (Xiaodong, 2018). Although methods such as stratification at varying temperatures (Kai et al., 2016), altering seed moisture by scarification (Yanfang et al., 2017), ultrasound treatment (Nong et al., 2012), and chemical or hormonal mutagenesis (Li et al., 2007) have been used to slightly alter the dormancy period of seeds and accelerate the germination process, a significant reduction in the dormancy period of *Paris polyphylla* var. *yunnanensis* seeds through genetic means has not yet been achieved. The fundamental mechanisms of seed dormancy in *Paris polyphylla* var. *yunnanensis* remain unclear.

Methods for shortening the seed dormancy period of *Paris polyphylla* var. *yunnanensis* reveals that treatments such as cold stratification at 4°C, altering seed moisture content, ultrasonic treatment, and chemical or hormonal mutagenesis (Lai et al, 2023) are all critical inducers of epigenetic changes in seed genes. Thus, it is hypothesized that modifying epigenetic markers in the seed genome could be an approach to significantly shorten the seed dormancy period of *Paris polyphylla* var. *yunnanensis*. However, research on the mechanisms underlying alterations in seed epigenetics as well as the epigenetic background and intrinsic regulatory patterns of seeds remains relatively insufficient.

Our previous research revealed that during the entire dehiscence period lasting over 20 days, the anthers of *Paris polyphylla* var. *yunnanensis* consistently closed when the evening temperature decreased and reopened as the morning temperature increased. Furthermore, darkness, low temperature, and humidity can trigger the closure of dehiscent anthers (Wang et al., 2008a, b). Transcriptomic data indicate that numerous genes related to seed germination and dormancy undergo expression changes in pollen, with their expression patterns highly resembling those of germinating seed embryos. Therefore, it has been postulated that the frequent closure of anthers may be associated with seed dormancy (Cheng et al., 2019). However, the regulatory effect of pollen on the dormancy or germination of genes in seeds remains unclear.

By conducting a comparative analysis of open and closed anthers, low-temperature treatment of pollen, and low-temperature treatment of seeds using reduced representation bisulfite sequencing (RRBS), we aimed to identify gene clusters susceptible to DNA methylation induced by low-temperature treatment. Furthermore, we sought to establish an analytical model of changes in gene expression from pollen treatment to seed dormancy by investigating the significance of artificially treated pollen on harvested seeds after artificial pollination during seed germination and dormancy. This study contributes to unveiling the potential patterns in the transition between seed dormancy and germination.

## Materials and methods

### Plant materials

The anthers and pollen grains of *Paris polyphylla* var. *yunnanensis*, along with all seeds used for the experiments, were sourced from the Xundian planting base of Yunnan Modu Agriculture Co., Ltd. (Shunxin Village, Beidaying Town, Xundian Hui, and Yi Autonomous County, Kunming City, Yunnan Province), situated at an altitude of 2,211 m with an average annual temperature of 23°C and an average annual rainfall of 935.1 mm. Transcriptome analysis of tissues surrounding the anthers and gynoecium of *Paris polyphylla* var. *yunnanensis* was analyzed using data from 2014 (**Table 1**). Biological replicates T1, T2, and T3 represent different time points within half an hour from the closed to the fully open state, whereas T4, T5, and T6 represent biological replicates at different time points within 45 min from the open to closed state, all derived from the same plant. T7 corresponded to ruptured anthers; normal tissues around the gynoecium included the leaves (T12), petals (T13), sepals (T14), and stigma (T15). All tissue samples were collected and immediately stored in liquid nitrogen at −80°C until RNA extraction (Wang, 2021).

**Table 1:** Sample materials and collection conditions for RNA-seq.

Name	Tissue	State
T1	Tapetum contain pollen	Anthers remain closed in the morning
T2	Tapetum contain pollen	Anther was opening but not completely in the morning
T3	Tapetum contain pollen	Anther was opened completely in the morning
T4	Tapetum contain pollen	Anther closure initiated in the evening
T5	Tapetum contain pollen	Anther was closing but not completely in the evening
T6	Tapetum contain pollen	Anther was closed completely in the evening
T7	Tapetum contain pollen	Anther open in the first time
T12	Leaf	Same plant with collected anther
T13	Petal	Same plant with collected anther
T14	Sepal	Same plant with collected anther
T15	Stigma	Same plant with collected anther

**Figure 1:**
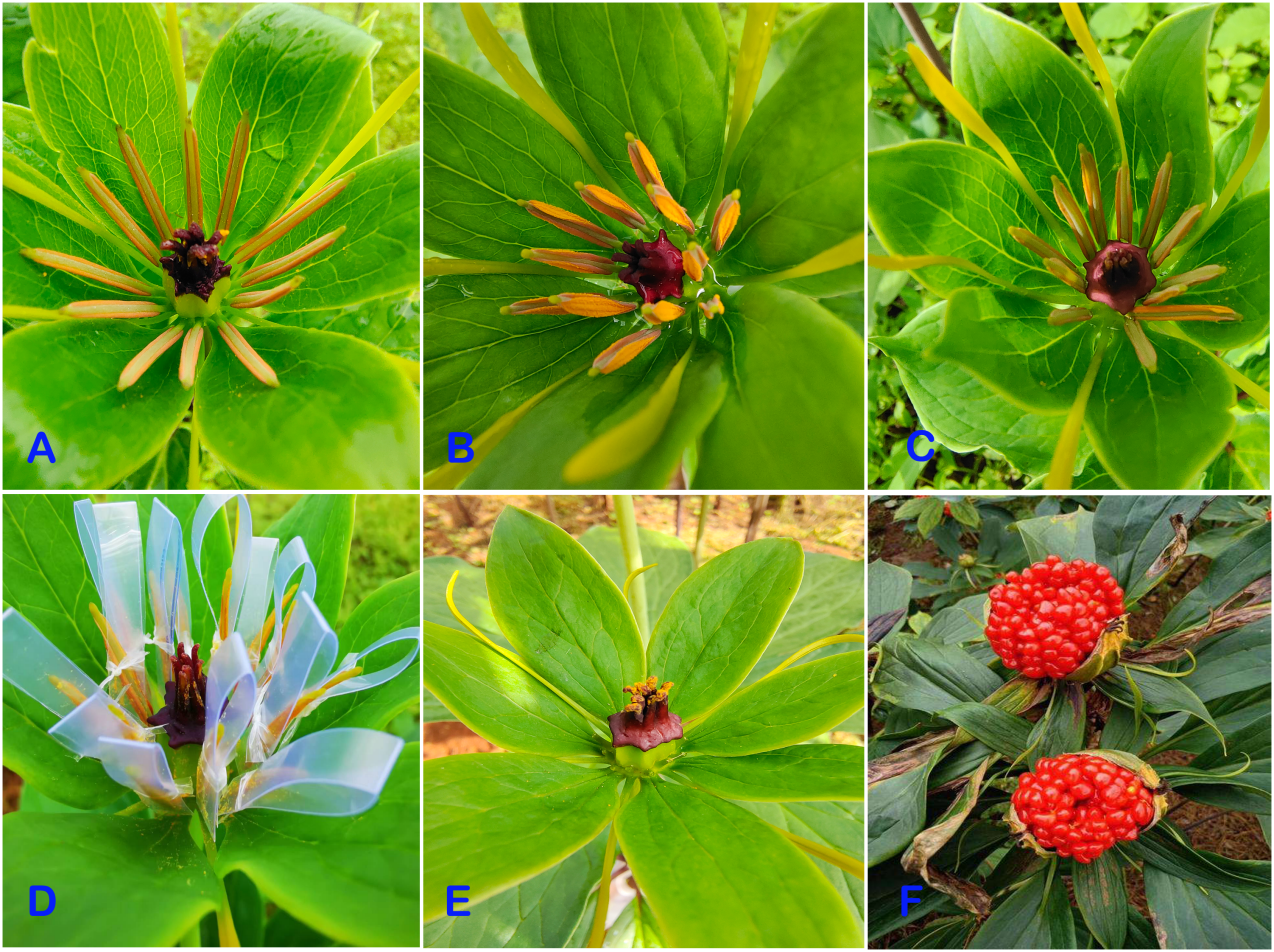
The diverse pollen conditions employed in the verification models and the subsequent creation of artificially pollinated seeds. **(A)** Pollen in unopened anther for model II. **(B)** Control: normal opening anther. **(C)** Control: normal closed anther after opening. **(D)** Model Ш: the close-block anther in order to keep “absolutely opening”. **(E)** Stigma after artificial pollination. **(F)**. Seeds obtained from artificial pollination of pollen.

The collected pollen was stored at 4°C (designated as 4-H) and 25°C (designated as 25-H), and the seeds were stored at 4°C (designated as 4-Z) and −85°C (designated as 85-Z) (**Table 2**). Seeds used for validation experiments included: control group, seeds produced by artificially isolated pollination of normal dehiscent anther on the 10th day; low-temperature protection model I: seeds produced by artificial isolation pollination of pollen from anthers after 2–3 days of dehiscence stored at 20°C for 72 h (pollen unaffected by low temperatures); closed anther model II: seeds produced by artificial isolation pollination of mature just-dehiscent anthers (in a fully closed state); and preventing anther closure model III: seeds produced by artificial isolation pollination of anthers that were prevented from closing using an artificial device for 20 days after 2–3 days of dehiscence (simulating an absolute open state), followed by pollination with isolated pollen. All seeds were artificially pollinated in June and harvested in November of the same year (**Table 3**, **Figure 1**).

**Table 2:** Cryopreserved methylome sequencing samples and preliminary processing techniques.

Code	Samples	Experimental methods	Treatment duration
4-H	Pollen	Keep at 4°C	30 days
25-H	Pollen	Keep at 25°C	30 days
4-Z	Seeds	Keep at 4°C	30 days
85-Z	Seeds	Keep at −85°C	30 days

**Table 3:** Experimental study on seed development models of pollen fertilization under various treatments.

Code	Pollen primordium state	Treatment procedures	Processing duration	Pollination process	Seed harvesting time
Control	Anther dehiscence on the third day	Keep pollen in anther open or close repeatedly	0 h	Artificial pollination	November of that year
Model I	Anther dehiscence on the third day	Keep pollen at 20°C	72h	Artificial pollination	November of that year
Model II	Anther dehiscence freshly	Collect pollen in just-dehiscent anther	0 h	Artificial pollination	November of that year
Model Ш	Anther dehiscence on the third day	Keep pollen in close-blocked anther	20 days	Artificial pollination	November of that year

### RNA extraction and library construction

Total RNA was extracted using an RNeasy Plant Kit (BioTeke, Beijing, China), and RNA samples were quantified using an Agilent 2100 bioanalyzer (Agilent Technologies, USA). The total RNA concentration for all samples exceeded or equaled 400 ng/μL; the OD260/280 ratios ranged between 1.8 and 2.2, with a 28S:18S ratio above 1.0 and RNA integrity numbers greater than 7.0. mRNA enrichment was performed using the NEBNext Poly(A) mRNA Magnetic Isolation Module, followed by cDNA library construction using the Illumina-specific NEBNext mRNA Library Prep Master Mix (New England Biolabs, USA) and NEBNext Multiplex Oligos (New England Biolabs, USA). Gel electrophoresis on a 1% agarose gel was used to assess the size of the library insert fragments, followed by real-time PCR quantification using the Library Quantification Kit from Illumina GA Universal (KAPA, USA). Qualified libraries underwent clustering on the Illumina cBot (Illumina Inc., USA) and were sequenced using the Illumina HiSeq 2500 (Biomarker Biotechnological Co., Beijing, China).

Raw paired-end reads were processed to obtain “clean reads” for *de novo* assembly, involving the removal of adapter contamination, unknown nucleotides, and low-quality reads with ambiguous sequences represented by “N”. Subsequently, Trinity software was used for *de novo* assembly of all clean reads obtained from multiple samples (Grabherr et al., 2011). Assembled reads were further consolidated into contigs representing longer transcripts, and primary transcripts or unigenes were selected based on pairing information and contig similarities. Unigenes were assembled using Bowtie (Langmead et al., 2009), and their expression levels were estimated using RNA-Seq by Expectation-Maximization (RSEM) software (Li and Dewey, 2011). Gene expression levels were determined by calculating RPKM values (reads per kilobase per million mapped reads) (Mortazavi et al., 2008). The RPKM method was used for gene expression quantification and was calculated using the following formula:


$$RPKM=\frac{total\;exon\;reads}{mapped\;read\left(million\right)\;*\;exon\;length\left(KB\right)}$$

The RPKM method mitigates the impact of variations in gene length and sequencing depth on the calculation of gene expression, enabling direct comparison of gene expression differences between different samples.

### Gene annotation and analysis

Functional annotation was carried out by aligning sequences against the NCBI NR (http://www.ncbi.nlm.nih.gov/), Swiss-Prot (http://www.expasy.ch/sprot/), COG (http://www.ncbi.nlm.nih.gov/cog/), and KEGG (http://www.KEGG.JP) databases using BLAST software. The functions were described using Gene Ontology terms (http://www.geneontology.org/) and classified using WEGO software (http://wego.genomics.org.cn/cgi-bin/wego/index.pl).

### Differential gene expression analysis

The EBSeq software (Leng et al., 2013) was used to identify genes with significant differences. The threshold for false discovery rate (FDR) is set at<0.01, and the fold change threshold is set at ≥2. the Benjamini–Hochberg method was used to correct p-values based on the FDR values. Differentially expressed unigenes were clustered using the UPGMA clustering method.

### Reduced representation bisulfite sequencing

Genomic DNA was extracted from the treated granules (**Table 2**) using a TIANamp Genomic DNA Extraction Kit (TIANGEN) according to the manufacturer’s protocol. Approximately 1 µg of genomic DNA was digested with MspI enzyme at 37°C for 16 h after mixing with the unmethylated lambda DNA. Purified DNA was processed using a MinElute PCR Purification Kit (Qiagen), followed by treatment with T4 DNA polymerase, Klenow fragment, and T4 polynucleotide kinase for end repair, A-tailing, and phosphorylation. Next, an adapter containing 5′-methylcytosine instead of cytosine was adenylated by Klenow fragment (3′–5′ exonuclease) and ligated using T4 DNA ligase to construct Illumina paired-end sequencing libraries. Unmethylated cytosines were converted to uracil using a Zymo EZ DNA Methylation-Gold Kit. Finally, PCR was performed in a 50-µL reaction volume, including 20 µL DNA with ligated adapters, 4 µL of 2.5 mM dNTPs, 5 µL of 10× buffer, 0.5 µL JumpStart Taq DNA polymerase, 2 µL PCR primers, and 18.5 µL water. The thermal cycling program for PCR consisted of an initial denaturation at 94°C for 1 min, followed by 12 cycles of 94°C for 10 s, 62°C for 30 s, 72°C for 30 s, and a final extension at 72°C for 5 min, with the products stored at 12°C. Size-selected libraries were analyzed using a Bioanalyzer system (Agilent, Santa Clara, USA), quantified via real-time PCR, and analyzed on the Illumina sequencing platform.

The raw data obtained were processed using the cutadapt tool to remove adapter sequences, with the parameters set to “-a AGATCGGAAGAGC -m 35 -n 2”. The cleaned read sequences were then mapped back to the genome using BSMAP software version 2.90 (Xi and Li, 2009), with parameters set to “-n 0 -v 0.08 -g 1”. Methylation rates were calculated using only uniquely aligned read sequences. Only cytosines with sufficient sequencing depth (greater than or equal to 5× coverage) in the CpG context were retained for further analysis. Differentially methylated regions (DMRs) were detected at CpG sites with at least 5× coverage using the *de novo* mode in methylene (Jühling et al., 2016). The parameters were set to “–mincpgs 5 –minMethDiff 0.1 –mtc 1–X 1–Y 1–v 0.7”. Subsequently, the detected DMRs were filtered based on the following criteria: (1) P-value less than 0.01, (2) methylation level difference greater than 0.2, (3) Number of CpGs included in the DMR greater than 5, and (4) length of DMR greater than 50 bp.

We analyzed the differential methylation regions in 4-H, 25-H, 4-Z, and 85-Z samples. For 4-H vs. 25-H, we examined differences in DNA methylation in CG-rich regions and promoter areas between the two samples. Similarly, in 4-Z vs. 85-Z, methylation distinctions were observed in CG-rich genomic regions and promoter sites.

### Quantitative PCR analysis

For quantitative PCR analysis, cDNA was reverse-transcribed from mRNA extracted from the four model plant seeds (**Table 3**) and used as the quantitative PCR template. Reverse transcription of cDNA was performed using an All-In-One 5X RT MasterMix kit (Shanghai Ruibose Biological Technology Co., Ltd.). qRT-PCR was performed using an Archimed X4 Medical Fluorescence Quantitative PCR Instrument (Kunpeng (Xuzhou) Scientific Instruments Co., Ltd.). The PCR primers were synthesized by Beijing Qiangke Biological Technology Co., Ltd. (Beijing, Beijing) (as shown in **Table 4**). The Taq Pro Universal SYBR qPCR Master Mix (Nanjing Novogene Biological Technology Co., Ltd.) was used.

**Table 4:** Primers for qRT-PCR analysis.

Primer name	Primer sequence F (5′–3′)	Primer sequence R (5′–3′)
ACT-2 (internal control primers)	CTCTCTCAGCACCTTCCAGCAG	TAACAACCCAAACAAACAATCC
ARP	TCCGTCTACGACTGGCTCTA	TTCACGAGTAGGACCACCGA
IAAH	CCCTTCGGGCTGATATGGAC	CCTTCCTCTGCGGGTTGAAA
SUS	AACGGTTTATGGAGGGCCAG	TAGATTCGTTGTAGCCCCGC
ABI2	AGATGGGCCCTTTGGTTCAG	CAAAACGTTTCCGCCCTTGT
GA2ox	GTCCGCTTATCCTCGAAGGG	AATCCCGGTGGTAGACCTGA

The qRT-PCR reaction system, with a total volume of 20 µL, consisted of 10 µL SYBR Taq enzyme, 4 µL cDNA (50 ng/µL), 2 µL Primer Mix (10 μM), and 4 µL nuclease-free water. The reaction protocol involved an initial denaturation at 95°C for 5 min, followed by 40 cycles of denaturation at 95°C for 15 s and annealing at 53°C for 15 s. Each treatment was performed in triplicate for independent replicates. Statistical analysis was conducted using the 2-(ΔΔCT) method and GraphPad Prism 8 software, applying the t-test for variance analysis.

## Results

### Comparative transcriptomic analysis of pollen and surrounding tissues

Post-RNA-seq sequencing, initial data quantity, and quality assessments revealed that all samples exhibited a Q30>93%. After filtering the raw data, reads with base counts below 20 were limited to under 20%, the N content did not surpass 5%, and ribosomal RNA was absent (**Table 5**). Using Trinity software (Trinity: https://github.com/trinityrnaseq/trinityrnaseq/releases), a unified gene database was constructed from mixed samples, encompassing 12,083,102 contigs, 280,777 transcripts, and 79,815 unigenes. The resulting transcripts had an N50 length of 1,215 bp, whereas the average length of the unigenes was 1,098 bp. Bowtie analysis indicated an alignment efficiency of >62.19% for each read.

**Table 5:** Quality assessment of transcriptome data.

SampleID	ReadSum	BaseSum	GC (%)	N (%)	Q20 (%)	CycleQ20 (%)	Q30 (%)
T1	16999694	3433596623	50.27	0.01	98.75	100.00	93.42
T2	16265835	3285366091	50.07	0.01	98.78	100.00	93.54
T3	15645762	3160147874	49.36	0.01	98.77	100.00	93.52
T4	15701026	3171292675	49.58	0.01	98.80	100.00	93.61
T5	16011697	3234047704	49.64	0.01	98.79	100.00	93.58
T6	15683182	3167681443	50.30	0.01	98.77	100.00	93.52
T12	13981216	2823955927	49.03	0.01	98.82	100.00	93.76
T13	14886624	3006832373	47.38	0.01	98.89	100.00	94.06
T14	14224023	2872983353	49.07	0.01	98.81	100.00	93.70
T15	14635771	2956134845	48.34	0.01	98.81	100.00	93.77

**Figure 2:**
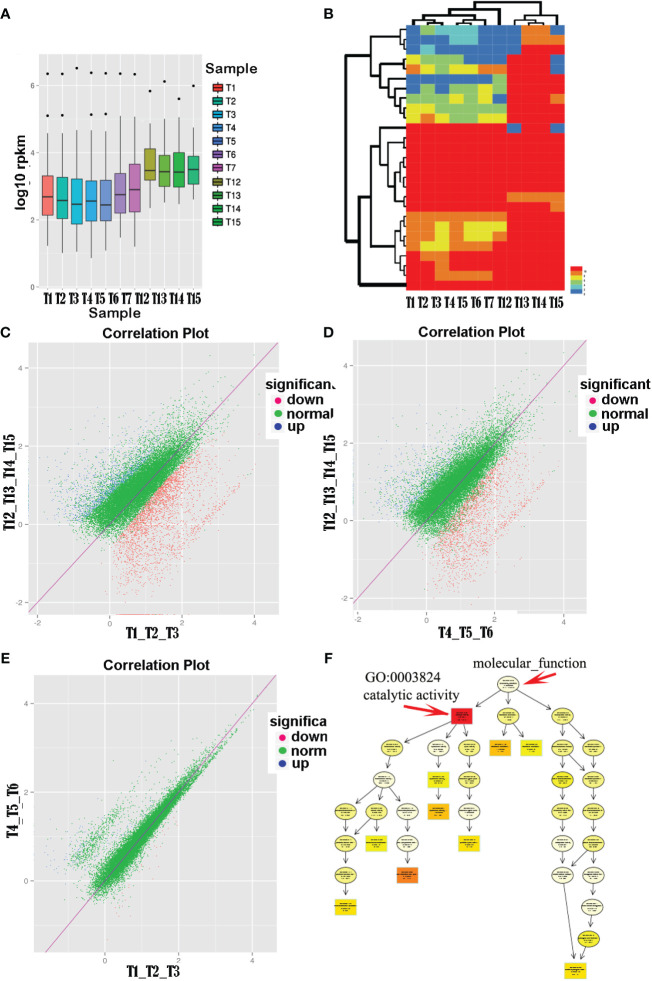
Comparison of pollen transcription levels in different states of anther groups with normal tissues. **(A)** Transcriptomic analysis revealed higher overall expression levels in normal tissues (T12–T15), including leaves, petals, sepals, and pistils, compared with pollen tissues (T1–T7). **(B)** Clustering analysis based on unigene’s relative expression levels (RPKM values). **(C–E)** Differential expression analysis comparing the open and closed anther groups with normal tissues showed a significant upregulation of pollen-related genes in closed anthers relative to open ones. **(F)** Functional enrichment analysis of differentially expressed genes between closed anthers and controls highlighted a significant enrichment of genes associated with catalytic activity (GO:0003824). The topmost term with the highest significance level in the directed acyclic graph was catalytic activity, comprising various specific enzyme categories.

Analysis of transcript abundance boxplots (**Figure 2A**) revealed a clear segregation into two groups (i.e., anther samples T1–T7 and floral organ samples T12–T15), with anthers showing significantly lower average gene expression levels than floral organs. Cluster analysis of the unigene expression profiles for each sample indicated two main branches: one representing the pollen group and the other representing normal tissues. Notably, within the pollen group, open anthers (T1, T2, and T3) and closed anthers (T4, T5, and T6) formed distinct subclusters (**Figure 2B**).

Further comparative analyses were conducted on the expression of unigenes in the open anther, closed anther, and normal control groups and between the open and closed anther groups. The results demonstrated a substantial upregulation of gene expression in both the open and closed anther groups compared with that in the normal control group. Specifically, when comparing open anther states to the controls (T1_T2_T3 vs. T12_T13_T14_T15), there were 2,830 differentially expressed genes, of which 2,077 (73.4%) were upregulated and 750 (26.6%) were downregulated. Similarly, when comparing closed anther states to controls (T4_T5_T6 vs. T12_T13_T14_T15), 1,991 genes were differentially expressed, with 1,625 (81.62%) upregulated and 66 (18.4%) downregulated genes in closed anthers.

A comparison between the open and closed anther groups (T1, T2, and T3 vs. T4, T5, and T6) revealed 136 differentially expressed genes, predominantly (107, 79%) exhibiting higher expression in closed anthers, whereas a smaller subset (28, 21%) displayed lower expression. In summary, there was a significant increase in gene expression in anthers compared with normal tissues, with a higher proportion of upregulated genes in closed anthers than in open anthers (**Figures 2C–E**).

Unigene sequences were aligned against a public database for functional annotation to further analyze the differentially expressed genes in anthers. The annotations were predominantly based on established model organisms, with 20% referencing *Vibrio vinifera* (grapevine), 6% *Oryza sativa* Japonica Group (japonica rice), and 5% *Theobroma cacao* (cacao). The remaining studies used *Ricinus communis* (castor bean), *Populus trichocarpa* (poplar), *Setaria italica* (foxtail millet), *Prunus persica* (peach), *Zea mays* (corn), *Sorghum bicolor* (sorghum), *Cucumis sativus* (cucumber), *Aureococcus anophagefferens* (brown tide algae), *Brachypodium distachyon* (purple false brome), and *Fragaria vesca* subsp. *Vesca* (wild strawberry), the *Oryza sativa* Indica Group (indica rice), *Glycine max* (soybean), and *Arabidopsis thaliana* (thale cress) accounted for less than 4%, whereas other species accounted for 30%. The results indicated that gene clusters that were significantly enriched for differential gene functions between closed anthers and controls corresponded to GO:0003824 catalytic activity, with subsequent levels including GO:0016740 transferase activity, GO:0016787 hydrolase activity, and GO:0016829 lyase activity (**Figure 2F**).

### Screening genes prone to DNA methylation changes in pollen and seeds

A Fisher’s exact test was used alongside the Benjamini–Hochberg multiple correction to functionally annotate the specific differentially expressed genes in the anthers of *Paris polyphylla* var. *yunnanensis*. Differential gene expression between the open anther groups and normal tissues was subjected to KEGG enrichment analysis. The results revealed that of the total assembled *Paris polyphylla* var. yunnanensis, Unigene 6630 was associated with KEGG pathways. Among these, 148 genes (2.23%) were enriched for plant hormone signal transduction (Ko04075). Comparing the DEGs between the open anther groups and normal tissues (T1_T2_T3 vs. T12_T13_T14_T15), 311 genes were linked to KEGG pathways. Within this set, 27 unigenes enriched in plant hormone signal transduction represented 8.68%, indicating significant enrichment (corr _ p-value = 7.6532e-08).

**Figure 3:**
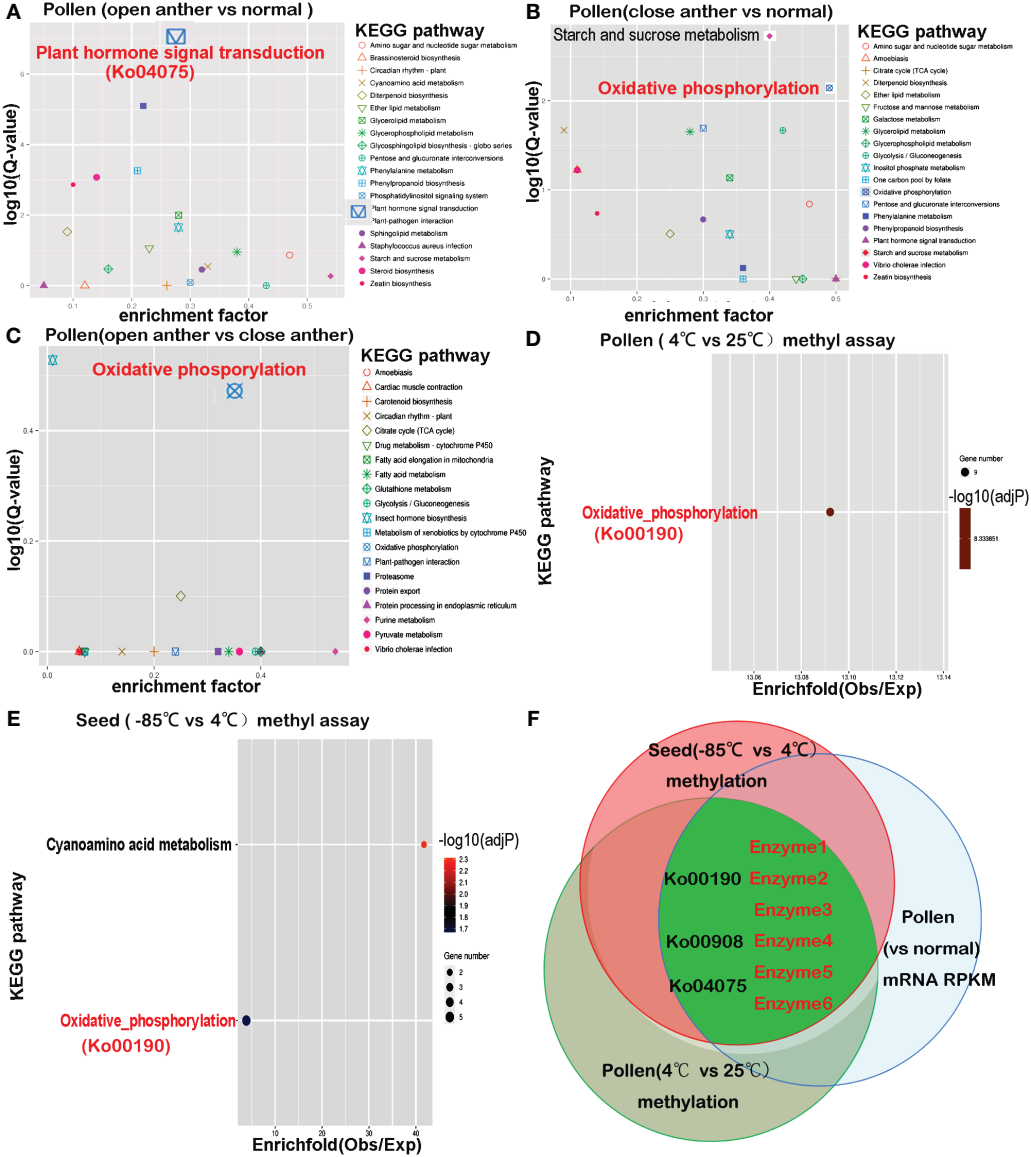
The KEGG pathways enriched in differentially expressed genes from transcriptome comparisons overlap with pathways enriched in differentially methylated genes. **(A)** Differential gene enrichment analysis between the open anther group (T1–T3) and normal tissues (T12–T15) reveals that the most significant KEGG pathway is plant hormone signal transduction (Ko04075). **(B)** Differential gene enrichment analysis between the closed anther group (T4–T6) and normal tissues (T12–T15) shows that the most significant KEGG pathways are starch and sucrose metabolism and oxidative phosphorylation (Ko00190). **(C)** Differential gene enrichment analysis between the open anther group (T1–T3) and closed anther group (T4–T6) highlights oxidative phosphorylation (Ko00190) as the most significant KEGG pathway. **(D)** Methylation differential gene enrichment analysis of anthers treated at 4°C and 25°C indicates that the most significant KEGG pathway is oxidative phosphorylation (Ko00190). **(E)** Methylation differential gene enrichment analysis of seeds treated at −85°C and 4°C identifies oxidative phosphorylation (Ko00190) as one of the most significant pathways. **(F)** The overlapping pathways enriched in transcriptome and methylation analyses reveal that key enzymes affecting pollen developmental gene expression undergo methylation changes due to decreased temperature, consistent with seed behavior. enzyme1: NADH-ubiquinone oxidoreductase chain 5; enzyme2: plasma membrane ATPase 4; enzyme3: cytochrome c oxidase subunit 2; enzyme4:cis-zeatin O-glucosyltransferase; Enzyme5: ABSCISIC ACID-INSENSITIVE 5-like protein 4; enzyme6: indole-3-acetic acid-amido synthetase.

Further analysis displayed that in the set of differentially expressed genes between open anther groups and normal tissues (T1_T2_T3 vs. T12_T13_T14_T15), 15 pathways remained highly significant after Ko04075, with a corrected p-value of<0.01. These included pathways such as plant–pathogen interaction (ko04626), steroid biosynthesis (ko00100), zeatin biosynthesis (ko00908), phenylalanine metabolism (ko00360), diterpenoid biosynthesis (ko00904), and phosphatidylinositol signaling system (ko04070), as well as energy metabolism-related pathways like phenylpropanoid biosynthesis (ko00940), glycerolipid metabolism (ko00561), ether lipid metabolism (ko00565), glycerophospholipid metabolism (ko00564), starch and sucrose metabolism (ko00500), sphingolipid metabolism (ko00600), amino sugar and nucleotide sugar metabolism (ko00520), cyanoamino acid metabolism (ko00460), and glycosphingolipid biosynthesis - globo series (ko00603) (**Figure 3A**).

Comparison between the differentially expressed genes in the closed anther group and normal tissues (T4_T5_T6 vs. T12_T13_T14_T15) showed that the oxidative phosphorylation pathway (ko00190) was most significantly enriched with 30 unigenes, followed by starch and sucrose metabolism (ko00500) with 25 unigenes, and glycolysis/gluconeogenesis (ko00010) with 20 unigenes. Other significantly enriched pathways included pentose and glucuronate interconversions (ko00040), diterpenoid biosynthesis (00904), glycerolipid metabolism (ko00561), *Vibrio cholerae* infection (ko05110), amino sugar and nucleotide sugar metabolism (ko00520), zeatin biosynthesis (ko00908), phenylpropanoid biosynthesis (ko00940), ether lipid metabolism (ko00565), inositol phosphate metabolism (ko00562), phenylalanine metabolism (ko00360), and citrate cycle (TCA cycle) (ko00020) (**Figure 3B**). Notably, ko00500, ko00190, ko00010, and ko00020 were key pathways in energy metabolism. Additionally, the pathways directly linked to plant hormone signaling pathways included 00904, ko00908, ko00940, and ko00360. The pollen transcriptomes of open- and closed-state anthers were compared (T1_T2_T3 vs. T4_T5_T6) to further investigate this disparity. The results revealed that out of the 41 unigenes associated with the KEGG pathways, seven were enriched in oxidative phosphorylation (ko00190) (corr_p_value = 3.4577e-01) and four were enriched in the citrate cycle (TCA cycle) (ko00020) (corr_p_value = 7.9357e-01) (**Figure 3C**), which are both critical pathways in energy metabolism.

RRBS methylation sequencing was used to analyze the whole-genome methylation of *Paris polyphylla* var. *yunnanensis* pollen and seeds. The constructed *Paris polyphylla* var. *yunnanensis* RRBS library was subjected to bisulfite treatment with a non-methylated C-to-T conversion rate exceeding 97%. Sequencing analysis showed that cold-treated pollen (4-H) and 25°C-treated pollen (25-H) yielded 46.68Gb and 34.22Gb data, respectively, whereas −85°C-treated seeds (85-Z) and 4°C-treated seeds (4-Z) yielded 71.76 Gb and 74.4 Gb data, respectively (**Table 6**).

**Table 6:** Quality control data of methylation sequencing results for low-temperature-treated pollen and seeds.

Sample	RawReadsNum	AdapterCleanBaseRate	CleanReadsNum	CleanBase Num	CleanQ20 Rate	CleanQ30 Rate	CleanData (Gb)	Conversion Rate
25-H	477995710	47.88%	473111408	34221305807	98.04%	95.00%	34.221	0.977301
4-H	467490740	66.68%	466251134	46678242359	97.72%	93.95%	46.678	0.983456
85Z	485553402	98.71%	485242920	71775076943	95.00%	87.75%	71.775	0.994134
4Z	505715868	98.21%	505353272	74401645969	95.48%	88.76%	74.402	0.993022

AdapterCleanBaseRate: Utilize the software “cutadapt” for filtering raw data, which involves trimming adapter sequences, removing bases with quality scores less than 5 from the beginning and end of sequences. Calculate the ratio of post-quality control bases to total bases in the raw data (CleanBase/RawBase). ConversionRate: During library construction, the evaluation of transformation efficiency can be enhanced by incorporating a segment of non-methylated DNA sequence, such as the lambda sequence. Theoretically, post sodium bisulfite treatment, all cytosine (C) sites should convert to thymine (T), resulting in 100% transformation efficiency. By quantifying the rate at which Cs in the lambda sequence convert to Ts (i.e., 1 − methylation rate), the actual transformation efficiency of the library can be determined.

A KEGG pathway intersection analysis was performed to compare methylation between 25°C- and 4°C-treated pollen, followed by comparison with −85°C- and 4°C-treated seeds to validate whether genes in pollen susceptible to methylation changes after low-temperature treatment may undergo methylation alterations in seeds. The results showed that the most significantly enriched KEGG pathway associated with genes showing differential DNA methylation between 4°C and 25°C treatments was oxidative phosphorylation (ko00190) (overall expect value 2.42, rich factor 4.12, multiple testing p = 0.0013) (**Figure 3D**). Other enriched pathways included a two-component system, plant hormone signal transduction, zeatin biosynthesis, RNA degradation, endocytosis, and amino and nucleotide sugar metabolism. For seeds treated at 4°C compared with those at −85°C, genes associated with differentially methylated DNA regions were most notably enriched in cyanoamino acid metabolism and oxidative phosphorylation (**Figure 3E**), with additional enrichment in plant hormone signal transduction, necroptosis, zeatin biosynthesis, endocytosis, RNA transport, alpha-linolenic acid metabolism, and other signaling pathways. The results showed that the intersecting pathways enriched for genes undergoing methylation changes in both cold-treated pollen and seeds included oxidative phosphorylation, plant hormone signal transduction, zeatin biosynthesis, and endocytosis.

A shared enrichment pathway for methylation was identified by subjecting pollen and seeds to low-temperature treatment. The intersection of this pathway with differentially expressed genes between anthers and normal tissues led to the discovery of three intersecting pathways: Ko00190, Ko00908, and Ko04075. Genes present in all three pathways were susceptible to methylation alterations. Among these, six genes directly overlapped (**Table 7**, **Figure 3F**) and encoded six key enzymes: enzyme1 in the Ko00190 pathway was NADH-ubiquinone oxidoreductase chain 5 (ND5); enzyme2 was plasma membrane ATPase 4 (AHA4); enzyme3 was cytochrome c oxidase subunit 2 (COX2) in the same pathway. In Ko00908, the overlapping enzymes were cis-zeatin O-glucosyltransferase (CISZOG1), and in Ko04075, the overlapping enzymes were ABSCISIC ACID-INSENSITIVE 5-like protein 4 (ABF1) and indole-3-acetic acid-amido synthetase (IAAS).

**Table 7:** Overlapping of Methyl-Diff genes and metabolic pathway in cold-treated seeds and pollen.

Name	DMR_geneNum	DMR_Hyper genebody	DMR_Hyper_promoter	DMR_Hypo genebody	DMR_Hypo_promoter
Oxidative phosphorylation (ko00190)	Pollen (4°C vs. 25°C)	NADH-ubiquinone oxidoreductase chain 5; plasma membrane ATPase 4		Cytochrome c oxidase subunit 2; NADH-ubiquinone oxidoreductase chain 5; plasma membrane ATPase 4	Cytochrome c oxidase subunit 2; NADH-ubiquinone oxidoreductase chain 5
	Seed (−85°C vs. 4°C)	Cytochrome c oxidase subunit 2; NADH-ubiquinone oxidoreductase chain 5; plasma membrane ATPase 4		Cytochrome c oxidase subunit 2; NADH-ubiquinone oxidoreductase chain 5	NADH-ubiquinone oxidoreductase chain 5
Zeatin biosynthesis (ko00908)	Pollen (4°C vs. 25°C)	Zeatin O-glucosyltransferase; cis-zeatin O-glucosyltransferase	Zeatin O-glucosyltransferase; cis-zeatin O-glucosyltransferase		
	Seed (−85°C vs. 4°C)	Zeatin O-glucosyltransferase	Zeatin O-glucosyltransferase		
Plant hormone signal transduction (ko04075)	Pollen (4°C vs. 25°C)			Indole-3-acetic acid-amido synthetase GH3.4; ABSCISIC ACID-INSENSITIVE 5-like protein 4	Indole-3-acetic acid-amido synthetase GH3.4; ABSCISIC ACID-INSENSITIVE 5-like protein 4
	Seed (−85°C vs. 4°C)	Indole-3-acetic acid-amido synthetase GH3.4; protein TRANSPORT INHIBITOR RESPONSE 1; two-component response regulator ARR2-like	Indole-3-acetic acid-amido synthetase GH3.4; Protein TRANSPORT INHIBITOR RESPONSE 1	Indole-3-acetic acid-amido synthetase GH3.4; protein TRANSPORT INHIBITOR RESPONSE 1; ABSCISIC ACID-INSENSITIVE 5-like protein 4	Indole-3-acetic acid-amido synthetase GH3.4; protein TRANSPORT INHIBITOR RESPONSE **1**; ABSCISIC ACID-INSENSITIVE 5-like protein 4

In the context of anther dehiscence inhibition, closed anthers exhibited reduced expression levels of enzyme1 and enzyme6, alongside increased expression levels of enzyme2, enzyme3, enzyme4, and enzyme5. Storing pollen grains at 4°C and subjecting seeds to −85°C low-temperature treatment decreased DNA methylation in the promoter regions of enzyme1, enzyme2, and enzyme3 genes, with localized DNA methylation increasing in certain gene regions. Conversely, enzyme4 displayed heightened DNA methylation in both promoter and gene regions. Enzyme5 and enzyme6 genes in 4°C-stored pollen grains demonstrated reduced methylation in both the promoter and gene regions, whereas seeds treated at −85°C showed decreased DNA methylation in both regions for enzyme5 but varied patterns of methylation for enzyme6 across different regions. Reduced methylation in promoter and gene regions typically enhances gene expression; thus, the modified methylation patterns in enzyme1, enzyme2, enzyme3, enzyme5, and enzyme6 due to low-temperature treatments resulted in elevated expression in pollen grains (**Table 7**).

In treated seeds, enzyme1, enzyme2, enzyme3, and enzyme5 showed increased expression, although the impact on enzyme6 was uncertain. However, enzyme4 experiences reduced expression in both low-temperature pollen grains and seeds. Alterations in the methylation levels of enzyme2, enzyme3, and enzyme5 corresponded with expression changes due to anther closure, whereas inconsistent expression alterations were observed in enzyme1, enzyme4, and enzyme6 compared with those induced by anther closure.

**Figure 4:**
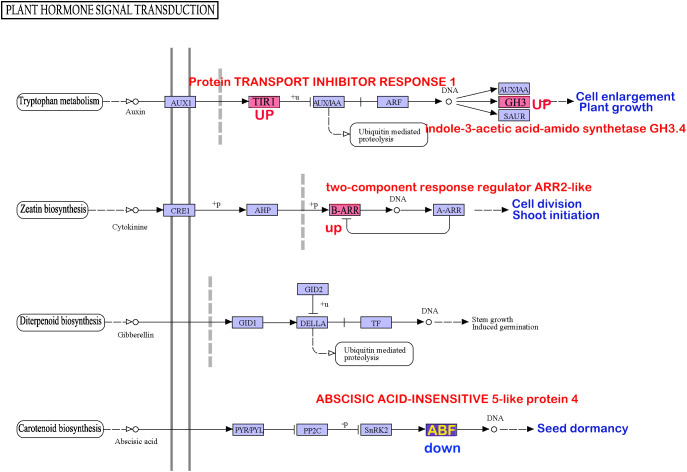
The genes prone to methylation alterations within pollen and seeds may influence seed dormancy through signaling pathways in agriculture and biology.

Analysis of the involvement of the six enzymes in both oxidative phosphorylation and plant hormone signal transduction pathways revealed intriguing insights. Enhanced expression of enzyme1, enzyme2, and enzyme3 potentially facilitates ATP production (**Supplementary Material Figure 1**). However, variations in enzyme5 and enzyme6 within the plant hormone signal transduction pathway may suppress seed dormancy and stimulate seed germination (**Figure 4**).

### Validating genes undergoing expression changes in pollen within fertilized seeds

We selected five genes with significant differential expression between anthers and normal tissues from transcriptomic analyses (sucrose synthase (SUS), auxin-repressed protein (ARP), IAAS, GA2OX (gibberellin 2-beta-dioxygenase), and ABI2) for analysis of expression pattern alterations to further elucidate the changes in the expression patterns of pollen in response to stimuli due to anther dehiscence.

**Figure 5:**
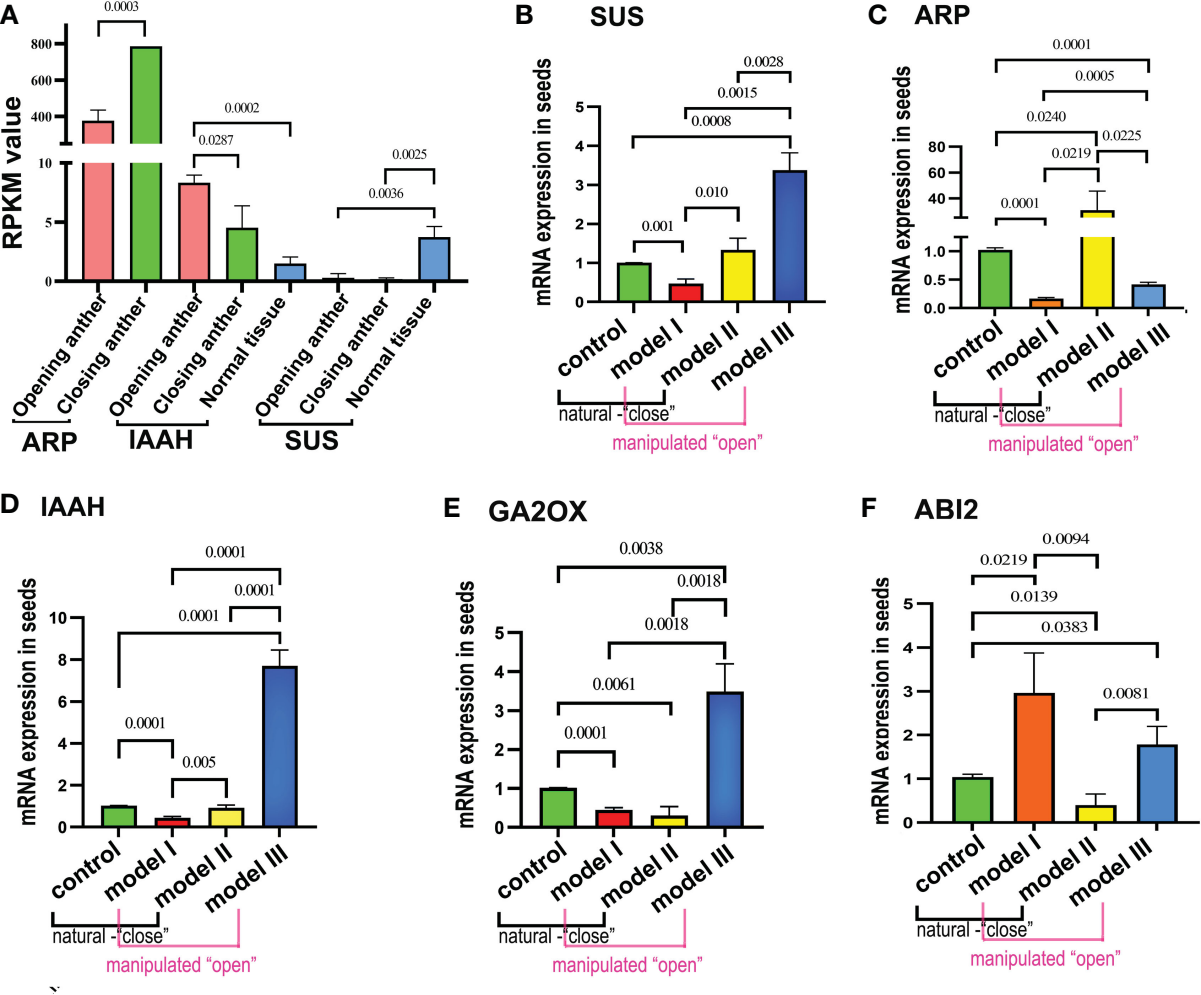
Validating gene expression disparities in the seed transcriptome of Polyphylla var. yunnanensis in anthers. **(A)** Transcriptome expression analysis reveals that the ARP gene exhibits high expression in closed anthers, whereas IAAS shows high expression in open anthers; SUS gene remains unchanged in expression during anther dehiscence. **(B)** The SUS gene demonstrates decreased expression in temperature protection model I and increased expression in open model Ш. **(C)** ARP displays a significant increase in expression in just-dehisced anthers of closed model II, whereas it shows decreased expression in models I and Ш. **(D-F)** IAAS and GA2OX genes exhibit elevated expression levels in model III and showing reduced expression in model I, meanwhile.GA2OX was also downregulated in model II.

Transcriptomic analyses revealed significant differences in the expression of ARP and IAAS, which are regulated by the opening and closing of physiological activities. ARP exhibited a notable difference in expression between the subtypes of open and closed anthers (p< 0.001), with higher expression in closed anthers. In contrast, IAAS showed a significant difference in expression between open and closed anthers (p< 0.05), with upregulation in open anthers and significantly higher expression in closed anthers than in normal tissues (p< 0.001). The expression of SUS remained unaffected by anther status, with reduced expression relative to that in normal tissues in the overall pollen (**Figure 5A**). GA2OX exhibited differences in expression between the closed anthers and normal tissues, whereas ABI2 displayed differential expression between the open anthers and normal tissues.

The expression levels of the five selected genes were analyzed in four groups of artificially pollinated seeds using qRT-PCR. The objective of this study was to observe any alterations in the expression patterns within the seeds of the remaining three groups compared with the control group. By comparing the expression patterns of these genes in the pollen transcriptome with the qRT-PCR results in seeds, the study aimed to investigate whether models I and III, characterized by “close state,” and models II and control, associated with “open state,” exhibited any changes in expression. Results indicated that the SUS gene displayed the lowest expression in seeds of model I, representing a cold protection model post-pollination at 20°C, and the highest expression in model III, which signifies inhibition of anther closing. Given the significantly low expression levels in both “open” and “close” state anthers in the transcriptome, it was determined that the expression trend of the SUS gene in the model III seeds did not align with the pollen transcriptome. However, the expression patterns in the seeds of the other three models were consistent with those in the pollen transcriptome. Upon examining the remaining three models based on the control group’s expression levels, a notable decrease in SUS gene expression was observed in model I seeds, whereas there was a significant increase in expression in model III seeds, along with an increase in model II as well (**Figure 5B**). The expression levels of the ARP gene were significantly lower in the cold protection model I and the closure prevention model III compared with the control group and closure model II. This finding is consistent with the pollen transcriptome expression pattern, in which the expression levels were low in open-state anthers and high in closed-state anthers. However, when comparing seed expression patterns, it was observed that the expression levels in models I and III were significantly reduced, whereas in model II, the expression levels were markedly increased (**Figure 5C**).

The IAAS gene showed the highest expression in model III while exhibiting the lowest levels in model I. Its expression pattern in model III aligned with the pollen transcriptome analysis, showing high expression in open-state anthers and low expression in closed-state anthers. However, it demonstrated a contrasting behavior in model I. Analysis of seed expression patterns also revealed significant upregulation in model III and downregulation in model I (**Figure 5D**).

Further analysis of two other key genes in the plant hormone signaling pathway, i.e., GA2OX (**Figure 5E**) and ABI2 (**Figure 5F**), showed that GA2OX undergoes expression pattern changes in models III, II, and I. Model III exhibited a significant increase in expression, whereas models I and II showed decreased expression. ABI2 showed increased expression in the seeds of models I and VII, whereas it was downregulated in model II.

## Discussion

### Cracked anthers closing and cold treatment boost energy metabolism

Comparative analysis of the transcriptomes of the tapetum and surrounding diploid anther tissues revealed distinct bifurcations, indicating a significant divergence in gene expression profiles between the entire anther and its neighboring tissues. Within the anther sample branch, the open and closed anthers were distinctly segregated into two subgroups. Additionally, “just cracked” anthers formed a third subclass, suggesting differential expression profiles even among the three anther subclasses. Although the overall expression levels in the surrounding anther tissues were higher than those in the anthers, examination of commonly expressed genes between the two groups revealed disparities in gene expression. Among the 2,830 genes compared between open anthers and surrounding tissues, the majority (73.4%) were highly expressed in open anthers (2,077 genes), with a small portion (26.6%) exhibiting lower expression levels. Similarly, when comparing “close state” anthers with surrounding tissues using 1,991 genes, a higher proportion of genes (81.62%) were upregulated in closed anthers (1,625 genes). When comparing the open anther subclass with the closed anther subclass, 79% (107 genes) of the 136 DEGs exhibited higher expression levels in the closed anther subclass. These outcomes suggested a specific set of anther genes with elevated expression relative to neighboring tissues, signifying a distinct group controlling anther closure physiology. Notably, among the two anther states, 107 genes were upregulated in closed anthers compared with open anthers, implying at least 107 genes regulate the physiological behavior of anther closure.

The most enriched pathways between open anthers and surrounding tissues were related to the plant hormone signaling pathway and energy metabolism. Conversely, in the pathways of differential gene enrichment between closed anthers and surrounding tissues, the two most enriched pathways were oxidative phosphorylation and starch and sucrose metabolism, which were primarily associated with energy metabolism. The differential gene enrichment pathways between open and closed anthers consisted entirely of energy metabolism pathways, including oxidative phosphorylation and the citrate (TCA) cycle. Thus, anther closure is mainly initiated and controlled by a group of genes that regulate energy metabolism.

The most significant gene-enriched pathway showing DNA methylation differentiation in pollen under the low-temperature treatment was oxidative phosphorylation (10 genes, p = 0.0013). Similarly, in seeds subjected to the low-temperature treatment, the most prominent pathways enriched with differentially methylated genes were cyanoamino acid metabolism and oxidative phosphorylation. The convergence of genes exhibiting methylation changes in both the pollen and seeds indicated an oxidative phosphorylation pathway. Considering that this pathway was also prominently enriched in differentially expressed genes from transcriptome comparisons between closed anthers and surrounding tissues, as well as open and closed anthers, it suggested that the opening and closing of anthers, along with low-temperature treatment of pollen and seeds, may activate energy metabolism-related genes that play a crucial role.

### Cold-induced methylation alterations in the genetic repertoire, potentially altering seed dormancy in pollen and seeds

Low temperatures are the primary cause of anther dehiscence (Wang et al., 2017), acting as a natural defensive mechanism in response to cold stimuli (Wang et al., 2008a). The induction of methylation changes in pollen genes responsible for anther protection by low temperatures implies that a subset of genes within the pollen genome are susceptible to cold-induced methylation. Consequently, these genes are likely to undergo methylation alterations in ex vitro pollen and seeds directly subjected to low temperatures. Moreover, among the differentially expressed genes in anthers compared with other tissues, a subset included genes affected by methylation changes.

The analysis revealed that the most significantly enriched molecular function of gene clusters showing specific expression in the anther transcriptomes, in contrast to other tissues, was GO:0003824, catalytic activity, indicating that these methylation-sensitive gene clusters likely belong to the enzyme category.

The intersection of significantly enriched differentially expressed genes in the transcriptomes from pollen treated at 4°C and seeds treated at −85°C revealed pathways shared across all three datasets. Notably, pathways, including oxidative phosphorylation (ko00190), zeatin biosynthesis (ko00908), and plant hormone signal transduction (ko04075), were concurrently enriched in all three gene sets. Examination of the overlap of genes enriched in the anther transcriptome, differentially methylated genes in pollen treated at low temperatures, and differentially methylated genes in seeds under low-temperature treatment within these pathways identified six enzymes that were consistent across all datasets. Low-temperature treatment resulted in uniform methylation changes in enzyme1, enzyme3, and enzyme5 in both anthers and seeds, which aligned with the expression changes observed during anther closure or opening. Conversely, enzyme2, enzyme4, and enzyme6 exhibited inconsistent expression trends compared with those influenced by anther closure or opening. Notably, among these six key enzyme genes, ND5, AHA4, and COX2 are critical enzymes in the oxidative phosphorylation pathway, and the ND5 protein is the core subunit of the mitochondrial membrane respiratory chain NADH dehydrogenase (complex I), which is considered essential for catalytic assembly. Complex I transfers electrons from NADH to the respiratory chain (Maldonado et al., 2020; Soufari et al., 2020; Klusch et al., 2023). AHA4 drives H+ co-transport, generating a proton gradient across the membrane and leading to external acidification and/or internal alkalinization, which may mediate growth responses. Most transport proteins in plant cells are energized by the action of the plasma membrane H+ pump fueled by ATP, which allows protons to traverse the electrochemical gradient of the membrane. AHA4 is crucial for plant growth and development, and phosphorylation of ATPase4 potentially served as a key regulatory factor in plant signal transduction processes (Palmgren, 2001; Haruta et al., 2015; Pei et al., 2022). Cytochrome c oxidase on the cell membrane links O2 reduction chemistry to proton translocation across membranes, aiding in generating an electrochemical proton gradient used to power ATP synthesis catalyzed by the rotary ATP synthase located within the same membrane (Wikström et al., 2018). COX2, a core subunit of mitochondrial cytochrome c oxidase, plays a pivotal role in various physiological processes.

The ABF protein interacts with the ABA-responsive element (ABRE) in plant hormone signal transduction, regulating the expression of numerous genes involved in abscisic acid (ABA) response (Choi et al., 2000). Reduced ABF expression diminishes the ability of plants to withstand environmental stressors, rendering them more susceptible. Within the plant hormone signal transduction pathway, decreased ABF levels can increase seed sensitivity, facilitating emergence from dormancy (Zhao et al., 2016). IAAS catalyzes the synthesis of indole-3-acetic acid (IAA)-amino acid conjugates, thereby preventing free IAA accumulation. Furthermore, the overexpression of IAAS-GH3-8 leads to abnormal plant morphology and growth delays (Xu et al., 2021), whereas silencing GH3.8 results in increased plant height (Sun et al., 2020).

Low-temperature treatment of anthers and seeds leads to the downregulation of energy metabolism-related genes, such as ND5, and the upregulation of AHA4 and COX2, promoting plasma membrane transmembrane transport and activating plant signal transduction pathways. This process may ultimately participate in seed dormancy and germination through hormone synthesis and regulation of plant hormone signal transduction pathways.

### Artificial pollen pollination might alter seed dormancy

Owing to the common physiological behavior of anthers dehiscing during pollen maturation, but only a small portion of anthers closing after dehiscence, the anther opening of *Paris polyphylla* var. *yunnanensis* is a relatively normal phenomenon, whereas its closure is a unique event. In our previous comparative transcriptome analysis, we found that anther closure in *Paris polyphylla* var. *yunnanensis* is more energy-consuming than the opening. Differential gene groups related to anther closure in *Paris polyphylla* var. *yunnanensis* were significantly enriched in genes associated with epigenetic changes, seed dormancy, or germination (Cheng et al., 2019). The purpose of this study was to focus on the impact of the unique physiological behavior of anther closure in *Paris polyphylla* var. *yunnanensis* on pollinated seeds. Therefore, using normally cyclic opening and closing anthers as controls, artificially preventing closure as a treatment represented “anther that never closes completely”. At this time, the pollen was only influenced by the open-state anthers, designated as model III. Additionally, to enhance the contrast, a group of “pollen absolutely unaffected by open-state anthers” was set up by taking mature just-dehisced anthers, denoted as model II, because the pollen at this time has been within closed flowers and hardly experienced the influence of dehiscent anthers. Furthermore, previous studies have found that the ecological factors influencing anther closure are mainly affected by temperature, light, and humidity changes (Wang et al., 2017). In Yunnan Province, anthers open when the temperature rises in the morning and close when the temperature drops in the evening, which are the main influencing factors for the daily opening and closure of anthers. Changes in light and humidity occur less frequently under natural conditions. Therefore, anther closure in the *Paris polyphylla* var. *yunnanensis* may play a role in protecting seeds from the effects of low temperature, light, and humidity. Hence, a group of pollen stored at 20°C avoiding light and constant humidity was established, referred to as model I.

Based on transcriptome analysis of anthers and methylation analysis of pollen and seeds under low-temperature treatment, it has been proposed that the opening and closing of anthers may result in a defense response to external stimuli, leading to epigenetic modifications in a set of genes. These modifications are speculated to be retained in the seeds by altering the expression patterns of seed genes, thereby influencing seed dormancy. Four different pollen treatment models were established, and changes in gene expression in harvested seeds after artificial pollination were analyzed to validate this hypothesis. In the four pollen treatment models, the control group used anthers with naturally open and closed states, preserving the effects of anthers being “open” or “closed”. Model I represented pollen from anthers just beginning to split and stored at a temperature of 20°C to simulate the midday temperature at the collection point of *Houttuynia cordata*, which was stored at 20°C to minimize the impact of low temperature, light exposure, and humidity changes, safeguarding them from potential harm. Model II involved collecting mature pollen from anthers that had just split open naturally, without being influenced by opening. Model III involved artificially preventing the closure of anthers after they were opened by subjecting the pollen in the anthers to continuous exposure to unprotected natural stimuli.

Through comparative transcriptome analysis of enriched anther closure-related genes, the expression patterns of five genes, SUS, ARP, IAAS, GA2OX, and ABI2, were found to be consistent in the closed state (open state vs. closed state) and in the embryo (endosperm vs. embryo), within the context of plant dormancy regulation (Cheng et al., 2019). These five genes were confirmed to be key regulators of dormancy (Qi et al., 2013). In other words, the selection criteria for these marker genes simultaneously fulfill three conditions. First, they are catalytic enzyme genes displaying significant expression differences between anthers and normal tissues in transcriptome analysis. Secondly, these genes belong to a group of genes prone to methylation, which was obtained from low-temperature-treated pollen and seed methylation analysis. These genes are associated with pathways related to plant hormone signaling and energy metabolism. Among these, SUS acts as a key enzyme in the energy metabolism pathways of starch and sucrose. ARP, IAAS, GA2OX, and ABI2 are crucial hormone regulatory genes in the plant hormone signaling pathway. Third, all five genes were directly linked to or regulated by genes associated with seed dormancy. Therefore, these five genes served as marker genes to analyze changes in expression patterns.

SUS plays a vital role in providing energy and carbon for the development of embryos and seeds in flowering plants. During seed dormancy, there is a significant accumulation of glucose, sucrose, and starch, and alterations in sucrose metabolism are mediated by SUS (Moreno Curtidor et al., 2020). Starch- and sugar-related indicators remained stable with increasing SUS enzyme content in long-dormant potato varieties. Conversely, the levels of reducing sugars and soluble starch enzymes increased in the relatively short-dormancy varieties (Liu et al., 2023). The elevation of SUS implies increased sucrose accumulation in seeds, resulting in reduced energy consumption for dormancy breakage (Yu et al., 2022); therefore, SUS elevation is unfavorable for dormancy release.

ARP, also known as dormancy-associated gene 1, is responsive to hormones involved in biotic stress defense responses, such as salicylic acid (SA) and methyl jasmonate (MeJA), as well as growth-regulating hormones, such as auxins. ARP serves as an excellent molecular marker gene for “dormancy”; when overexpressed in rice, it can inhibit cell separation, block the cell cycle, delay axillary bud germination, and modulate auxin signaling as a growth suppressor affecting rice development (Souza et al., 2019; Chen et al., 2023).

GA2ox participates in the regulation of gibberellin metabolism and signal transduction and influences plant height, dormancy release, flower development, and seed germination (Liu et al., 2022). Comparative proteomic analysis of mature dormant and germinating seeds of *Paris polyphylla* var. *yunnanensis* revealed that GA2ox is downregulated at the mRNA or protein level in germinating seeds compared with mature dormant seeds (Ling and Zhang, 2022). ABI2 acts as an inhibitor of the abscisic acid (ABA) signaling pathway and is crucial for regulating ABA-mediated seed dormancy. ABI2 represents a potential target for the redox regulation of plant hormone signaling pathways (Meinhard et al., 2002).

Alterations in the expression patterns of these genes in seeds after pollination were evaluated using the two reference systems. One system utilized a control model in seeds as the baseline reference, termed the control; the other system compared the expression patterns of genes in models I and III with an “open state” attribute and models II and the control with a “closed state” attribute, in relation to transcriptome analysis during pollen in “open state” anthers and “closed state” anthers, referred to as the transcriptome reference system. When utilizing the transcriptome reference system, the SUS gene exhibited decreased expression in model I and increased expression in model III seeds. When compared with the control, there was significant upregulation in closed model III seeds, reduced expression in model I seeds, and increased expression in model II seeds. For the ARP gene, no changes in expression patterns were observed using the transcriptome reference system. In contrast, analysis against the control indicated a significant decrease in expression levels in models I and III but a notable increase in model II. In the case of the IAAS gene, the expression pattern matched the transcriptome results with the reference system in model III but was reversed in model I. However, within the seed control, an increase was evident in models III and II. Two pivotal genes, GA2OX and ABI2, lacked conditions for comparison within the transcriptome and were analyzed against the control. GA2OX displayed markedly increased expression in inhibitory anthers in model III and reduced expression in the 20°C stored pollen model I and just-open anther of model II. In contrast, ABI2 expression increased in models I and III seeds and was downregulated in model II. Consequently, the prevention of anther closure, storage at 20°C, and utilizing freshly dehiscent anthers without closure states would all lead to altered dormancy gene expression patterns in subsequent seeds.

In conclusion, the expression levels of SUS, IAAS, GA2OX, and ABI2 were upregulated in seeds subjected to pollen fertilization with inhibited anther closure, with only downregulation observed in ARP gene expression. This trend likely promoted dormancy. Conversely, storing the fertilized seeds at 20°C led to decreased expression of dormancy-related genes, such as SUS, ARP, GA2OX, and IAAS. In contrast, the expression of ABI2 genes was upregulated, thereby facilitating seed germination. Upon pollination with fresh anther-dehiscent pollen, the expression of the dormancy-regulating genes SUS and ARP increased, and that of GA2OX and ABI2 decreased.

## Data availability statement

The original contributions presented in the study are publicly available. This data can be found here: https://www.ncbi.nlm.nih.gov/sra/PRJNA1090394.

## Author contributions

BW: Writing – review & editing, Writing – original draft, Validation, Supervision, Project administration, Methodology, Funding acquisition, Conceptualization. MW: Writing – original draft, Investigation, Data curation. QW: Data curation, Formal analysis, Funding acquisition, Validation, Software, Writing – review & editing. XW: Data curation, Project administration, Writing – review & editing. DW: Supervision, Data curation, Project administration, Writing – review & editing. XY: Investigation, Data curation, Project administration, Writing – review & editing. YQ: Investigation, Data curation, Project administration, Writing – review & editing. YD: Investigation, Data curation, Project administration, Writing – review & editing. MM: Investigation, Data curation, Project administration, Writing – review & editing.

## Supplementary material

Supplementary Figure 1 The genes prone to methylation modifications regulate energy metabolism via oxidative phosphorylation pathways.
