# Supplementary figures and images for: Exploring the potential of Paris polyphylla var. yunnanensis pollen manipulation in modifying seed dormancy
Source: Front Plant Sci. 2024 May 22;15:1389357. doi: 10.3389/fpls.2024.1389357 (PMC11152045; doi:10.3389/fpls.2024.1389357)

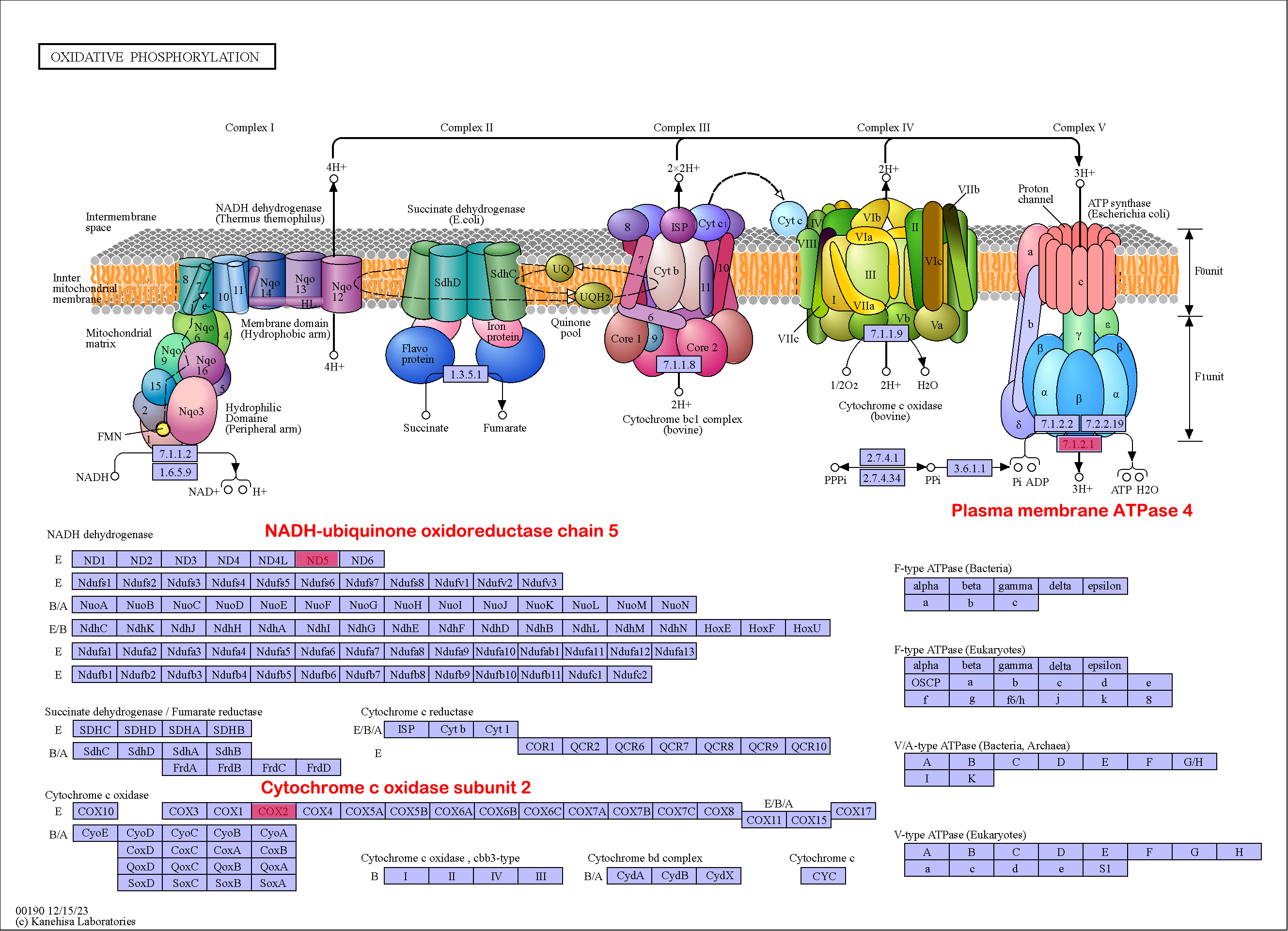

Supplement: Supplementary Figure 1 — The genes prone to methylation modifications regulate energy metabolism via oxidative phosphorylation pathways. [file Image_1.png]
